# Reconfigurable sound anomalous absorptions in transparent waveguide with modularized multi-order Helmholtz resonator

**DOI:** 10.1038/s41598-018-34117-z

**Published:** 2018-10-24

**Authors:** Houyou Long, Ying Cheng, Xiaojun Liu

**Affiliations:** 1 Key Laboratory of Modern Acoustics, Department of Physics and Collaborative Innovation Center of Advanced Microstructures, Nanjing University, Nanjing, 210093 China; 2 State Key Laboratory of Acoustics, Institute of Acoustics, Chinese Academy of Sciences, Beijing, 100190 China

## Abstract

Helmholtz resonators offer an ideal platform for advanced sound absorbers, but their utility has been impeded by inherent frequency range limitations and the lack of function reconfiguration. Here, we introduce a multi-order Helmholtz resonator (MHR) that allows multiple monopolar resonant modes theoretically and experimentally. The combination of these modularized MHRs further creates reconfigurable multi-band anomalous absorbers in a two-port transparent waveguide while maintaining undisturbed air ventilation. In asymmetric absorption state through coupling of artificial sound soft boundary with preposed MHR, sound energy is almost totally absorbed in multiple frequency ranges when sound waves are incident from one side while it is largely reflected back from the opposite side. Interestingly, the original asymmetric absorber would turn into symmetric bidirectional absorber if one post MHR concatenates after the soft boundary. Using combination of identical MHRs, we demonstrate function selective asymmetric/symmetric absorber in multi-bands, highlighting the potential to use MHRs in the design of diverse devices for more versatile applications.

## Introduction

Owning to the excellent characteristics of manipulating low-frequency sound waves with subwavelength dimension, the acoustic metamaterials based on traditional Helmholtz resonator (THR) have emerged as an attractive option in various fields such as sound proofing^1,2^, asymmetric sound transmission^3^, sound metadiffusers^4^, and acoustic superlens^5,6^ *et al*. However, many challenges have impeded the in-depth implementation of THR-based devices, including the frequency range limitations of strongly resonant devices, as well as adding reconfiguration to the devices. For example, the THR supports only one monopolar resonant mode and the constructed functional devices work at a single narrow band. Thus, the absorber based on identical THRs demonstrates only one absorptive peak^7–13^, let alone the function reconfiguration. A wide range of solutions to the operation frequency range problem have been presented in the literatures^9,14,15^, which involve the incorporation of many different THRs into the metamaterial devices and the combination of their respective frequency ranges. Additionally, many tuning techniques also span from active piezoelectric elements introduced into the THRs^16^ to passive constituent membranes^17^ that change acoustic response under specific tension stimuli. All approaches are at the cost of increasing fabrication complexity and expense.

In this work, we mitigate the above limitations posed by the operational frequency range and reconfiguration effectively through the proposed multi-order Helmholtz resonator (MHR). The MHR demonstrates multiple monopolar resonant modes which exhibit effective negative bulk modulus. Based on these identical modularized MHRs shunted in a double-port open waveguide, reconfigurable anomalous sound absorbers are constructed which can function as asymmetric and symmetric absorber, respectively. In the asymmetric system, the sound waves impinged from one port are nearly perfectly absorbed while from another port are largely reflected. The asymmetric absorptions originate from the anomalous reflections dominated by matched/mismatched surface impedances since the transmission are reciprocal. We emphasize that differing from the previous scheme^18–20^ which requires two slightly detuned resonators, the artificial soft boundary constructed here are based on identical MHRs. Therefore, the anomalous absorptions have been extended to multiple bands due to the advanced characteristics of the multi-band resonances of MHR. This asymmetric absorber can be conveniently reconfigured into a symmetric bidirectional absorber which can absorb the sound waves impinged from the both sides effectively.

## Results

### Multi-order Helmholtz resonator

**Figure 1:**
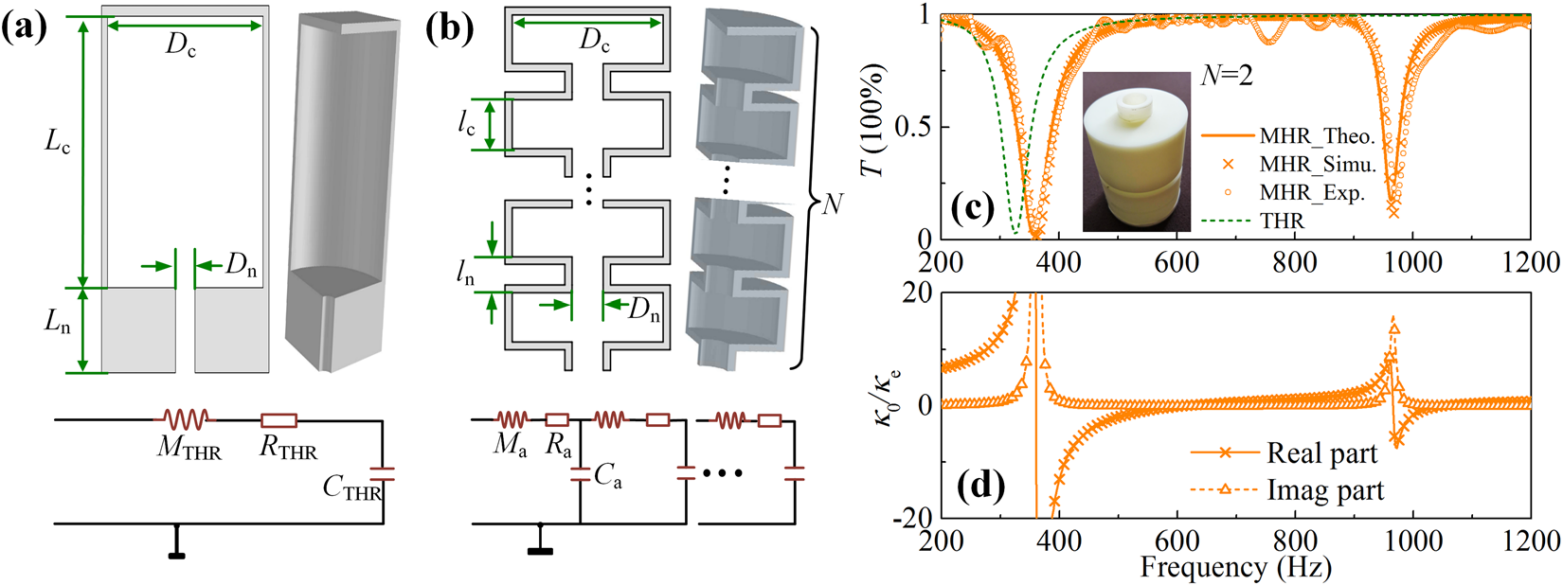
2D cross-section and 3D view of **(a)** the THR and **(b)** the MHR (a quarter of the complete structure). The insets show the corresponding effective electric circuit models. **(c)** The transmittances of the MHR with *N* = 2 (the orange solid line) and the THR (the olive dashed line) for reference. **(d)** The effective bulk modulus of the MHR.

We propose a type of variant Helmholtz resonator which can generate multi-order resonances while retaining monopolar characteristic. Figure 1(a,b) show the schematic view of the THR^2^ and the proposed MHR, respectively. The MHR is constructed by neck-and-cavity sub-structures of *N* elements arranged in a cascade way. *D*_n_ and *D*_c_ are the diameters of the cylindrical necks and cavities; *l*_n_ = *L*_n_/*N* and *l*_c_ = *L*_c_/*N* are their lengths. According to the lumped-parameter theory, the TMR and MHR can be equivalent to a spring-oscillator model depicted by the analogous electric circuit in the insets of Fig. 1(a,b), respectively. For THR, the neck is equivalent to the acoustic inductor with ${M}_{{\rm{THR}}}={\rho }_{0}{L}_{n}/{S}_{n}$ and the cavity is equivalent to the acoustic capacitor with ${C}_{{\rm{THR}}}={S}_{c}{L}_{c}/{\rho }_{0}{c}_{0}^{2}$, where ${S}_{{\rm{n}}}=\pi {D}_{{\rm{n}}}^{2}/4$ and $\,{S}_{{\rm{c}}}=\pi {D}_{{\rm{c}}}^{2}/4$. The corresponding equivalent parameters of MHR are ${M}_{{\rm{a}}}={M}_{{\rm{THR}}}/N$ and ${C}_{{\rm{a}}}={C}_{{\rm{THR}}}/N$. Additionally, the viscosity and radiation losses produced by the intensive scattering at resonance function as effective viscous and radiated resistor. Therefore, we can qualitatively elaborate that the MHR with *N* neck-and-cavity sub-structures could induce *N* discrete resonant modes. The quantitative analysis are as follows: taking the viscothermal losses into account, the complex wave number and impedance in the cylindrical tubes^21,22^ can be expressed as ${k}_{k}=\frac{\omega }{{c}_{0}}({\rm{1}}+\frac{\beta }{{s}_{{\rm{k}}}}(1+(\gamma -{\rm{1}})/\chi )$ and ${Z}_{{\rm{k}}}=\frac{{\rho }_{0}{c}_{0}}{{S}_{{\rm{k}}}}({\rm{1}}+\frac{\beta }{{s}_{{\rm{k}}}}({\rm{1}}-(\gamma -{\rm{1}})/\chi ))$, where ${{\rm{s}}}_{{\rm{k}}}(={r}_{{\rm{k}}}/\delta )$ is a defined parameter and ${S}_{{\rm{k}}}(\,=\,\pi {r}_{{\rm{k}}}^{2})$ is the area of corresponding tube (k = n for the neck and k = c for the cavity). $\delta =\sqrt{2\mu /{\rho }_{0}\omega }$ is the thickness of viscous boundary layer with *μ* = 1.568 × 10^−5^ m^2^/s being the viscosity of air. $\chi =\sqrt{{\rm{\Pr }}}$ with Pr (=0.702) is the Prandtl number under standard atmospheric pressure, $\beta =(1-j)/\sqrt{2}$ (*j* is the imaginary unit), and $\gamma ={\rm{1.4}}$ is the heat capacity ratio of air. Thus, the impedance of the MHR can be obtained as ${Z}_{{\rm{MHR}}}={M}_{{\rm{total}}}(1,1)/{M}_{{\rm{total}}}(2,1)$, where *M*_total_ is the total transfer matrix relating the sound pressure and velocity at the entrance and terminal of the MHR (see Methods for details).

In order to demonstrate the resonant characteristics of the MHR, we take a sample MHR with two layers of neck-and-cavity sub-structures for illustration (see inset of Fig. 1(c)), and the transmittance of MHR shunted in a straight transparent waveguide is shown in Fig. 1(c). The geometric parameters are listed in Table 1 (Sample 1). The theoretical (orange solid line), simulated (orange crosses) and experimental (orange circles) results are of good consistence. It is seen that there present two transmission valleys at 356 Hz and 963 Hz corresponding to the two resonant modes of the MHR, which confirm the theoretical prediction. For comparison, the transmittance of the THR with doubled neck length and cavity length is also plotted by olive dashed line and only one resonant mode is supported. Hence, the MHR exhibits improved abundant modes than the THR, and the number of modes is determined by the resonant order *N*. Moreover, the retrieved effective bulk modulus of the MHR (see Fig. 1(d)) turns negative at resonances, implying that all the resonances are in monopolar modes.

**Table 1:** Geometric parameters of the MHRs.

	*D*_n_ (mm)	*l*_n_ (mm)	*D*_c_ (mm)	*l*_c_ (mm)	*N*
Sample 1	13	11	50	29	2
Sample 2	15.4	11	50	19	4

### Reconfigurable sound absorbers

**Figure 2:**
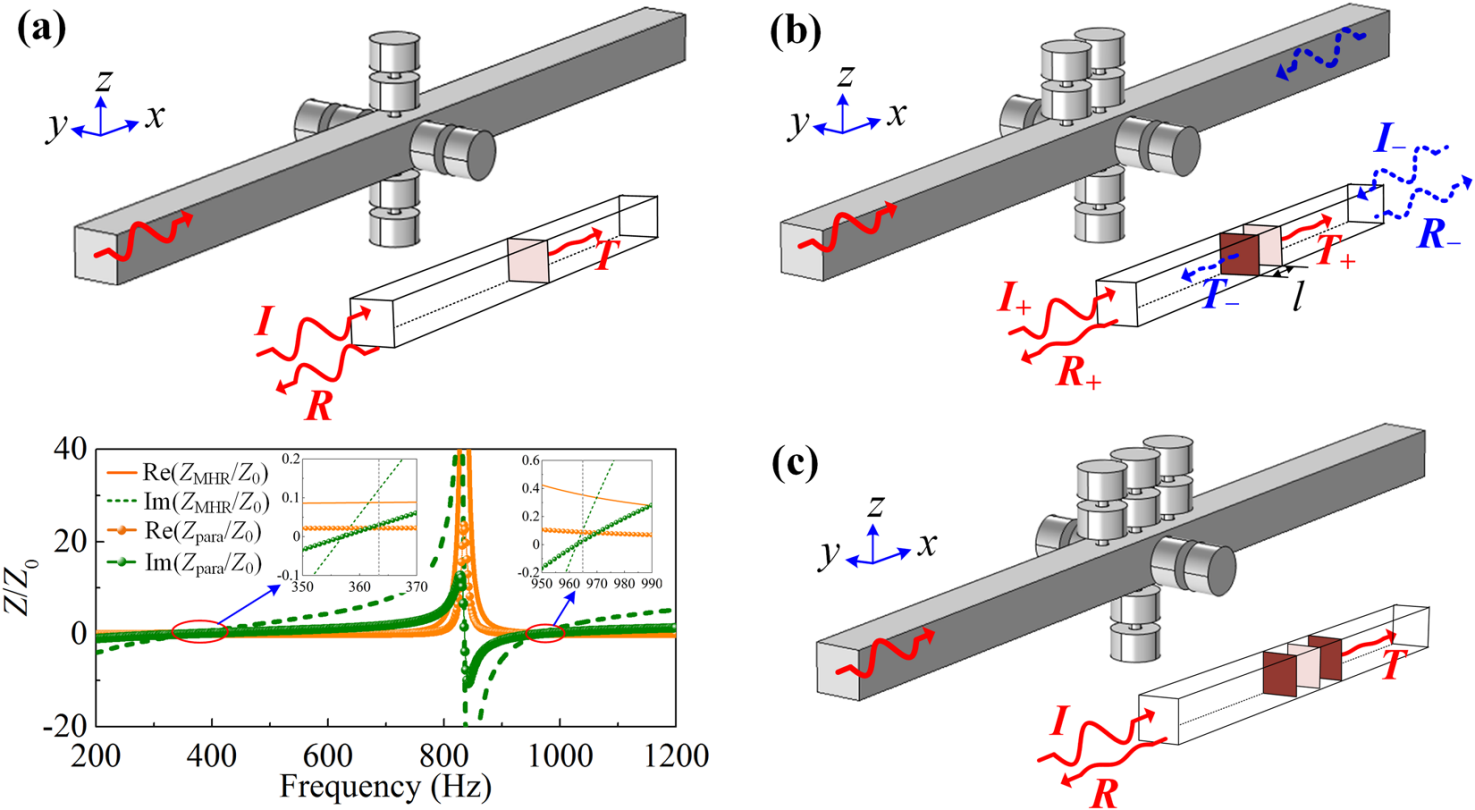
**(a)** 3D view of the artificial sound soft boundary (as sketched by the brown surface) constructed by parallel connecting four MHRs in a straight transparent tube and the impedance of the MHR (*Z*_MHR_) and parallel-mounted MHRs (*Z*_para_). **(b)** and **(c)** are corresponding for the 3D views of the asymmetric absorber and symmetric absorber.

Furthermore, we achieve reconfigurable anomalous absorption in a *transparent* acoustic waveguide by implementing the proposed modularized MHRs. We start from the effective soft boundary formed by mounting four parallel MHR in the waveguide sides, as shown in Fig. 2(a). The four parallel-mounted MHRs is equivalent to a virtual ultrathin surface with impedance ${Z}_{{\rm{para}}}={Z}_{{\rm{MHR}}}/4$ (sketched by the brown surface in the inset of Fig. 2(a)), where the relative impedances *Z*_para_ and *Z*_MHR_ normalized by air are plotted by lines and symbols, respectively. Note that *Z*_para_ = (0.02–0.005*i*)*Z*_0_ = (8.3–2.49*i*) kg/m^2^s at the resonant frequency of 356 Hz while *Z*_para_ = (0.09–0.004*i*)*Z*_0_ = (37.35–1.66*i*) kg/m^2^s at 963 Hz, which are far smaller than that of air (*Z*_0_ = 415 kg/m^2^s), as shown in the insets. Thus, the parallel-mounted MHRs can serve as a “soft” effective boundary at the two resonant frequencies. In addition, the system functions as a point symmetric scatterer, and the absorptance is constrained at 0.5 due to the continuity of sound pressure at the impedance surface^10^, i.e., the absorptance are 0.25 and 0.48 at the corresponding resonant frequencies. To break the limitation, a single MHR is mounted before the parallel-mounted MHRs as shown in Fig. 2(b). The system functions as an asymmetric absorber: (1) when sound waves impinge from the left port (as sketched by *I*_+_ in the inset), the sound energy are neither transmitted (${T}_{+}\to 0$) nor reflected (${R}_{+}\to 0$), and the system produce resultant near-perfect absorptance instead. (2) On contrary, the sound waves radiated from the right port (*I*_-_) has little transmittance (${T}_{-}\to 0$) but large reflectance (${R}_{-}\to 1$), and the absorption in this case is weak consequently. Here, the subscripts (+/−) stand for the propagation directions, i.e., “+” is for sound waves radiating from the left port (the red arrow) and “−” from the right port (the blue arrow). Furthermore, by connecting another MHR symmetrically behind the soft boundary as shown in Fig. 2(c), the original asymmetric absorptive system turns into a mirror symmetric one. Due to the shared soft boundary at the symmetric center, the system can efficiently absorb the sound energy radiated from both ports.

Transfer matrix analyses are further employed to demonstrate the reconfigurable extraordinary absorptions of the systems quantitatively. For the asymmetric system depicted in Fig. 2(b), the sound pressures and velocities in the input port and the exit port can be related by the transfer matrixes $T={T}_{{\rm{MHR}}}{T}_{{\rm{tube}}}{T}_{{\rm{para}}}$, where $${T}_{MHR}=[\begin{array}{ll}1 & 0\\ 1/{Z}_{{\rm{MHR}}} & 1\end{array}]$$, $${T}_{{\rm{Para}}}=[\begin{array}{ll}1 & 0\\ 1/{Z}_{{\rm{para}}} & 1\end{array}]$$ and $${T}_{{\rm{tube}}}=[\begin{array}{ll}\cos (kl) & j{Z}_{0}\,\sin (kl)\\ j\,\sin (kl)/{Z}_{0} & \cos (kl)\end{array}]$$ are the transfer matrixes of MHR, parallel-mounted MHRs and tube section. Here *l* is the distance between preposed MHR and the soft boundary. After some algebra, the pressure transmission and reflection coefficients are expressed as

1 $$t=\frac{2{e}^{jkd}}{{T}_{{\rm{11}}}+{T}_{{\rm{12}}}/{Z}_{0}+{T}_{{\rm{21}}}{Z}_{0}+{T}_{{\rm{22}}}}$$

2 $${r}_{+}=\frac{{T}_{{\rm{11}}}+{T}_{{\rm{12}}}/{Z}_{0}-{T}_{{\rm{21}}}{Z}_{0}-{T}_{{\rm{22}}}}{{T}_{{\rm{11}}}+{T}_{{\rm{12}}}/{Z}_{0}+{T}_{{\rm{21}}}{Z}_{0}+{T}_{{\rm{22}}}}.$$

3 $${r}_{-}=\frac{-{T}_{{\rm{11}}}+{T}_{{\rm{12}}}/{Z}_{0}-{T}_{{\rm{21}}}{Z}_{0}+{T}_{{\rm{22}}}}{{T}_{{\rm{11}}}+{T}_{{\rm{12}}}/{Z}_{0}+{T}_{{\rm{21}}}{Z}_{0}+{T}_{{\rm{22}}}}.$$

Therefore, the absorptances can be obtained as ${A}_{+}={\rm{1}}-|{r}_{+}{|}^{2}-|t{|}^{2}$ and ${A}_{-}={\rm{1}}-|{r}_{-}{|}^{2}-|t{|}^{2}$, respectively. Similarly, in the symmetric system shown in Fig. 2(c), the total transfer matrix turns into $T={T}_{{\rm{MHR}}}{T}_{{\rm{tube}}}{T}_{{\rm{para}}}{T}_{{\rm{tube}}}{T}_{{\rm{MHR}}}$ and the corresponding coefficients can be achieved by equations (1–3) as well. In this case, *r*_−_ equal to *r*_+_ since the elements of the transfer matrix satisfy the relationship of *T*_11_ = *T*_22_, which leads to the identical absorptances from the two ports consequently.

#### Asymmetric absorber

**Figure 3:**
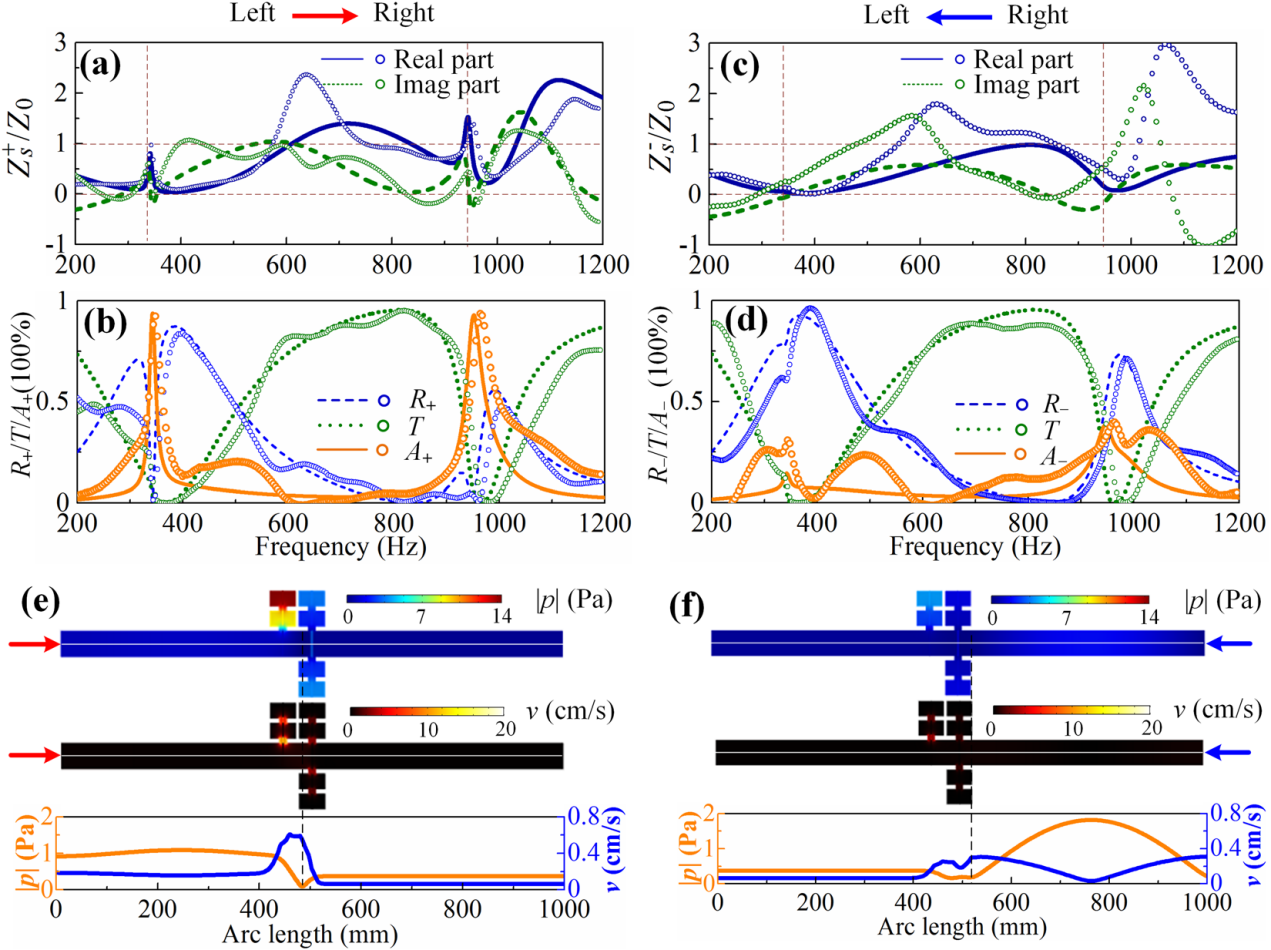
Normalized surface impedance of **(a)** the left side and **(c)** the right side of the asymmetric absorber where lines and symbols are for the theoretical and experimental results, respectively. **(b**–**d)** The corresponding reflectance (blue dashed lines/symbols), transmittance (olive dotted lines/symbols) and absorptance (orange solid lines/symbols). **(e)** and **(f)** are for the sound pressure and velocity when sound waves radiate from the left and right port, respectively.

Considering the reciprocal transmittance in the system, the asymmetric absorptions are dominated by the asymmetric reflectance expressed as ${R}_{\pm }=|({Z}_{s}^{\pm }-{Z}_{0})/({Z}_{s}^{\pm }-{Z}_{0}){|}^{2}$, where ${Z}_{s}^{\pm }$ is the surface impedances deduced from the transfer impedance formula and the superscripts (+/−) stand for the propagation directions (left/right). Figure 3(a) shows the surface impedance of the case when sound waves impinging onto the system from the left port, in which the lines and symbols are for the theoretical and experimental results. It is seen that the impedance tends to match that of air at resonances and the system has achieved the near-zero reflectance, i.e., 1.5% at 342.6 Hz and 3.3% at 952 Hz. Meanwhile, the transmittances are also near zero (4.6% and 3.6% at the corresponding frequencies) and consequently, the nearly-perfect absorptance can be achieved as presented in Fig. 3(b). The theoretical (experimental) absorptances are 93.9% and 93.1% (92.5% and 93.8%), respectively. In this case, the little reflection is due to the well-matched surface impedance with that of air and the near-zero transmission is supported by the soft boundary. However, when sound waves radiate from the right port, the impedances approaches zero as shown in Fig. 3(c) and the sound waves are largely reflected by the system with the reflectance of 80.9% and 61% at corresponding resonant frequencies. As a result, the theoretical (experimental) absorptances are minute to be 14.5% and 35.4% (30.7% and 36.6%) as demonstrated in Fig. 3(d). Thus, an asymmetric absorber in a two-port transparent straight waveguide with air ventilation has been built. Moreover, due to the near-zero reciprocal transmittances, the system also plays the role of a bidirectional sound insulator. We emphasize that the asymmetric absorber here is due to the hybridization of the bright mode (produced by the four parallel-mounted MHRs) and dark mode (induced by the single preposed MHR)^23^, which is physically different from the ones relying on the hybridizations of the two slightly detuned dark modes in previous works^19,20^. (see *Section Discussion* for detailed comparison).

In order to illustrate the mechanism of asymmetric absorption intuitively, the sound pressure |*p*| and velocity |*v*| distributions at the first peak (342.6 Hz) are depicted in Fig. 3(e,f), respectively. In the case of sound radiation from the left port, the sound energy is largely localized (top panel of Fig. 3(e)) and consumed in the preposed MHR as seen from the velocity distribution (middle panel of Fig. 3(e)). The sound pressure node and velocity antinode distribution at the central line of the waveguide (bottom panel of Fig. 3(e)) confirms that a soft boundary is formed near the parallel-mounted MHRs. However, when sound waves are radiated from the right port, just a small part of sound energy is trapped by the system and little energy is attenuated as seen from Fig. 3(f). Moreover, the right half tube presents the characteristics of strong standing wave as seen from the |*p*| profile, which implies that the sound pressures are largely reflected. In summary, the parallel-mounted MHRs physically behave a ‘virtual’ backed wall.

#### Symmetric absorber

**Figure 4:**
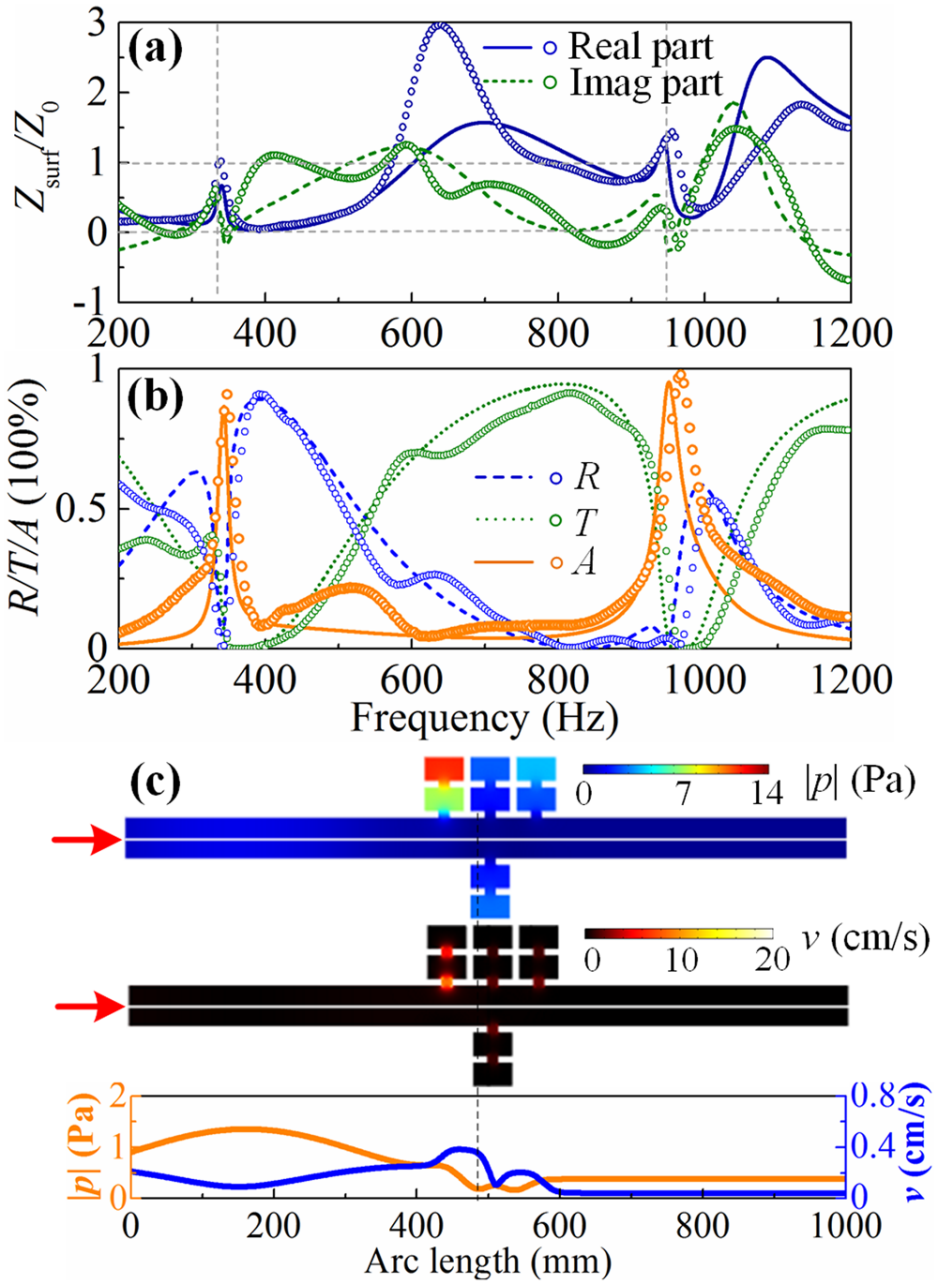
**(a)** Normalized surface impedance, **(b)** reflectance (blue dashed line/symbol), transmittance (olive dotted line/symbol) and absorptance (orange solid line/symbol) of the symmetric absorber. **(c)** The sound pressure and velocity distributions.

Similarly, the normalized surface impedance of the symmetric absorber (see Fig. 2(c) for schematics) is shown in Fig. 4(a), which matches that of air at resonances. Therefore, the reflected sound waves are almost cancelled and the absorptances of 86.7% and 95.3% at 344 Hz and 951 Hz in theoretical (90.9% and 97.9% at 348 Hz and 966 Hz in experimental) are achieved, as shown in Fig. 4(b). Figure 4(c) shows the sound pressure and velocity distributions in the symmetric system (take the case of sound waves incident from the left port for example) at the first absorptive peak. The sound energies are largely localized in the left MHR (top panel) since the soft boundary (bottom panel) block the sound waves to transmit though the right half tube. The sound energy has been attenuated in the left preposed MHR as seen from the velocity distribution (middle panel). Therefore, the right rear MHR makes no substantive influences on the absorptances and the symmetric absorptive system can be viewed as the combination of two asymmetric absorbers with a shared soft boundary.

### Four-band anomalous absorbers

**Figure 5:**
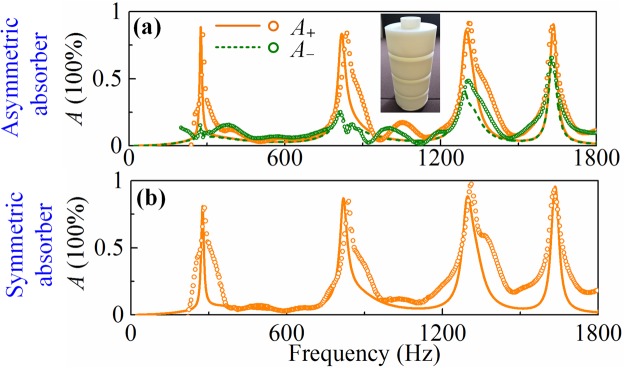
Multi-band absorptances of the **(a)** asymmetric and **(b)** symmetric system using MHR block with *N* = 4.

Since the proposed reconfigurable absorptive systems rely on the identical modularized MHRs, multi-band asymmetric and symmetric absorptions can be achieved if the resonator can support multiple resonant modes. Here, we employ the MHR with four layers of neck-and-cavity sub-structures and the parameters are listed in Table 1 (Sample 2). The four-band asymmetric absorptions are shown in Fig. 5(a) where the orange solid line is for the absorptance when sound waves radiating from the left port and the olive dashed line for radiating from the right port. The absorptive peaks are 88.5%, 85.5%, 86.3%, 90.7% at 276 Hz, 819 Hz, 1300 Hz, 1634Hz in theoretical analysis (82.6%, 85.2% 91.3% and 91.9% in experimental) when sound waves radiating from the left port, while reduced to 13.2%, 24.1%, 36.7%, 60.2% (15.2%, 20.4%, 48.1%, 63.2% in experimental) when radiating from the right port. Figure 5(b) shows the four-band absorption in symmetric system. The theoretical (experimental) peak values are correspondingly 81.2%, 87.1%, 88.3%, 95.7% in simulations (82.1%, 85.6%, 96.5%, 93.2% in experiments). Hence, the multi-band asymmetric and symmetric absorptions have been achieved.

## Discussion

We also note that the design and fabrication of advanced sound absorbers, which exhibits superior characteristics than conventional sound absorber such as acoustic porous materials^24^, has attracted considerable interest recently. To efficiently absorb the low-frequency sound energy with (deep−) subwavelength structure, a significant number of resonators have been employed to build up sound absorbers spanning from acoustic membrane^17,25,26^, acoustic metasurface^27,28^, acoustic split-ring resonator^29,30^, Mie resonator^31^, to labyrinthine Fabry-Perot resonator^32,33^, *et al*. However, most absorbers are based on single-port systems which need a rigid-backed wall to block the sound energy. Although such single-port absorbers can attenuate the sound energy, they also prevent air ventilation or light through. Additionally, the coherent perfect absorber (CPA) which demonstrates excellent absorptive performances in a common double-port system has been proposed^11,34,35^. However, the CPA requires two incident waves with strictly modified amplitudes and phases radiating from the two ports simultaneously. Hence, such CPA are rarely used to absorb sound energy but to be phase detectors^34,35^. Thus, we emphasize that the sound absorbers demonstrated here are in double-port structure with air ventilation while need no coherent incidences. These capabilities are valuable in applications such as simultaneously attenuate the noise and dissipate the heat produced by the machines.

**Figure 6:**
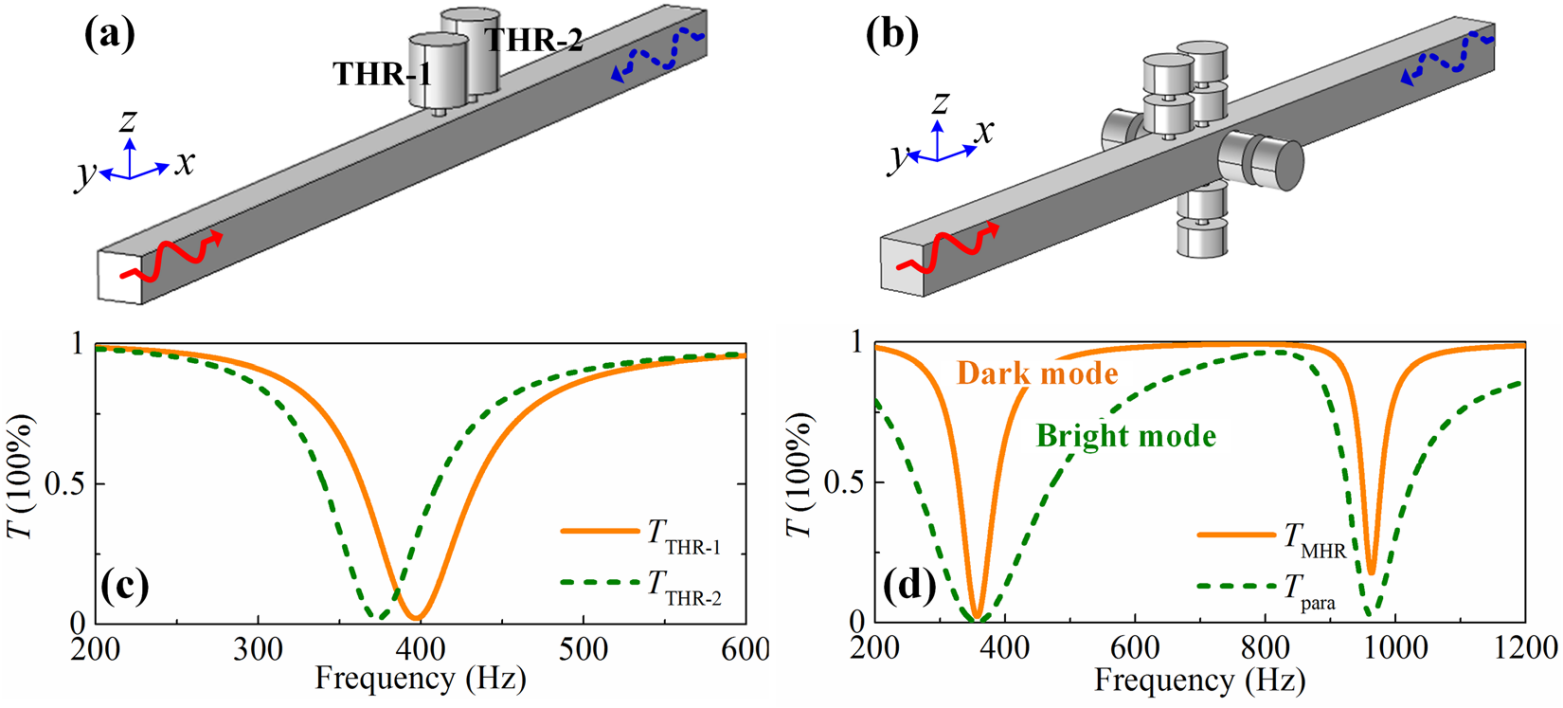
**(a)** 3D view of the asymmetric absorptive system presented in ref.^19^ and **(b)** in this work (also see Fig. 2(b)). (**c**) and (**d**) are the transmittances of corresponding systems.

In summary, we have designed a MHR exhibiting improved abundant modes than the THR and made up an artificial soft boundary with four parallel-mounted MHRs. An asymmetric absorber has been constructed by hybridizing the soft boundary acting as a bright mode with the single MHR acting as a dark mode, which is physically different from previous work^19^ [see Fig. 6(a)]. For clarity, the transmittances of the constructive blocks of the corresponding systems are illustrated in Fig. 6(c,d), respectively. The parameters of the Helmholtz resonators shown in Fig. 6(a) are listed in ref.^19^ and the MHRs in Fig. 6(b) are identical with the Sample 1. As seen from Fig. 6(c), the resonant frequencies of THR-1 and THR-2 are 397 Hz and 373 Hz, respectively, while the two THRs have almost the same quality factors $(Q={f}_{r}/{\rm{\Delta }}{f}_{r}$) since *Q*_THR-1_ = 5.7 and *Q*_THR-2_ = 5.3. Therefore, the two THRs are dark modes at different frequencies. However, the unit cells of the asymmetric absorber in this work [in Fig. 6(b)] have identical resonant frequency while different quality factors as seen from Fig. 6(d). Take the first resonant mode as an example, the quality factors are *Q*_MHR_ = 5.7 and *Q*_para_ = 1.5 which indicates that the single MHR acts as a dark mode while the parallel-connected MHRs as a bright mode according to the coupling-modes theory^23^. Therefore, two asymmetric absorbers have different coupling mechanisms. We also emphasize that the soft boundary may become even “softer” if more MHRs are parallelly connected, i.e., parallel mounting *N* (*N* > 4) MHR in a cylindrical waveguide, and the effect of asymmetric absorptions can be further strengthened.

Moreover, by further cascade a MHR after the parallel-mounted MHRs, the asymmetry breaks and the asymmetric absorber turns into a symmetric absorber conveniently. Owing to the characteristics of the multiple resonant modes of the MHR, the working frequencies are extended to multiple bands. The multiband absorption characteristics may prompt potential applications in noise control engineering, i.e., to eliminate the low-frequency noises in harmonic frequencies arisen from machines, cars and the high performance servers. The asymmetric and symmetric absorber can not only control sound absorption flexibly, but also allow the exchange of other mediums such as airflow. Furthermore, considering the multiple subwavelength resonant characteristics, the MHR can be utilized to devise versatile acoustic devices such as multi-band sound filter, sound retroreflector and topological phononic crystals, *et al*.^36,37^.

## Method

### Theoretical model of MHR

**Figure 7:**
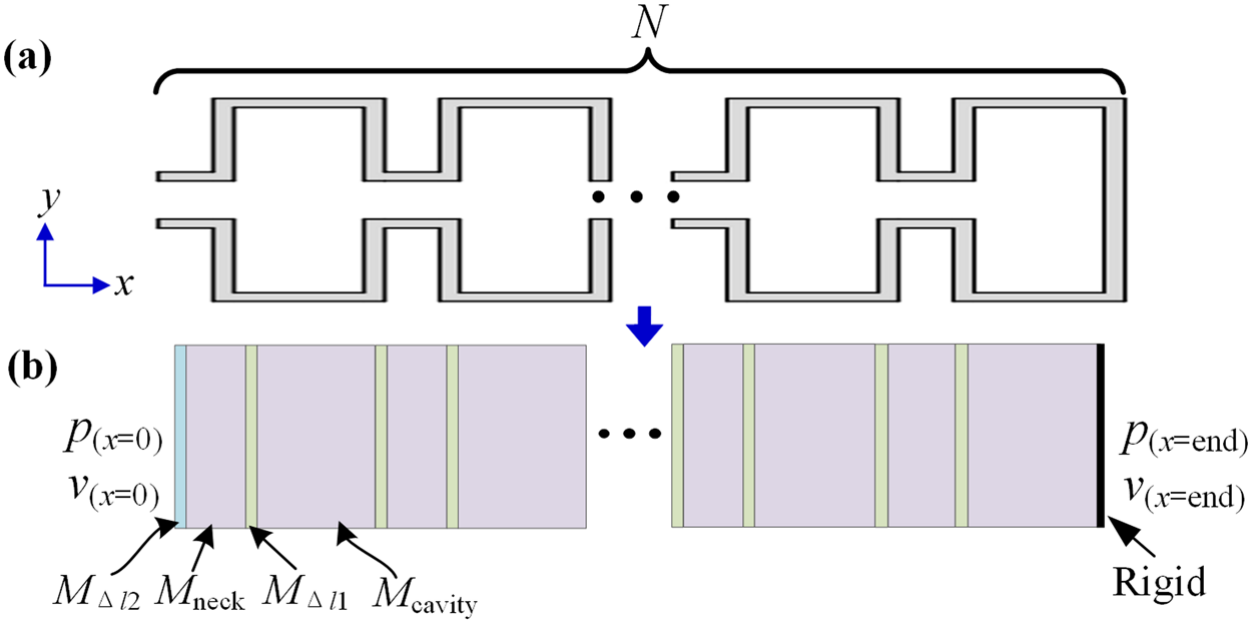
(**a)** 2D cross section of the MHR and **(b)** its effective model.

The theoretical analysis of the MHR is performed by using the transfer matrix method (TMM). Figure 7(a,b) show the 2D schematics of the MHR and the effective model, respectively. The relation between sound pressure and velocity at the neck of the MHR and that at the end of the MHR is expressed as:

4 $$[\begin{array}{c}{p}_{(x=0)}\\ {v}_{(x=0)}\end{array}]={M}_{{\rm{\Delta }}l2}{M}_{{\rm{neck}}}{M}_{{\rm{\Delta }}l1}{M}_{{\rm{cavity}}}{({M}_{{\rm{cell}}})}^{{\rm{N}}-{\rm{1}}}[\begin{array}{c}{p}_{(x=\mathrm{end})}\\ {v}_{(x=\mathrm{end})}\end{array}]={M}_{{\rm{total}}}[\begin{array}{c}{p}_{(x=\mathrm{end})}\\ {v}_{(x=\mathrm{end})}\end{array}]$$

Here, $${M}_{{\rm{cell}}}={M}_{{\rm{\Delta }}l1}{M}_{{\rm{neck}}}{M}_{{\rm{\Delta }}l1}{M}_{{\rm{cavity}}}$$. $${M}_{{\rm{neck}}}=[\begin{array}{cc}\cos ({k}_{{\rm{n}}}{l}_{{\rm{n}}}) & j{z}_{{\rm{n}}}\,\sin ({k}_{{\rm{n}}}{l}_{{\rm{n}}})\\ j\,\sin ({k}_{{\rm{n}}}{l}_{{\rm{n}}})/{z}_{n} & \cos ({k}_{{\rm{n}}}{l}_{{\rm{n}}})\end{array}]$$ is the matrix for the neck. $${M}_{{\rm{cavity}}}=$$$$[\begin{array}{cc}\cos ({k}_{{\rm{c}}}{l}_{{\rm{c}}}) & j{z}_{{\rm{n}}}\,\sin ({k}_{{\rm{c}}}{l}_{{\rm{c}}})\\ j\,\sin ({k}_{{\rm{c}}}{l}_{{\rm{c}}})/{z}_{{\rm{c}}} & \cos ({k}_{{\rm{c}}}{l}_{{\rm{c}}})\end{array}]$$ is the matrix for the cavity, where *z*_n_ and *k*_n_ are the effective impedance and wave number of the neck, *z*_c_ and *k*_c_ are the effective impedance and wave number of the cavity. *l*_n_ and *l*_c_ are the length of neck and the cavity. $${M}_{{\rm{\Delta }}l1}$$ and $${M}_{{\rm{\Delta }}l2}$$ stand for the matrixes derived from the pressure radiations at the discontinuity from the neck to the cavity and discontinuity from the neck to tube, which are expressed as $${M}_{{\rm{\Delta }}l1}=[\begin{array}{cc}1 & j{z}_{{\rm{n}}}k{\rm{\Delta }}{l}_{1}\\ 0 & 1\end{array}]$$ and $${M}_{{\rm{\Delta }}l2}=[\begin{array}{cc}1 & j{z}_{{\rm{n}}}k{\rm{\Delta }}{l}_{2}\\ 0 & 1\end{array}]$$, respectively. $${\rm{\Delta }}{l}_{1}$$ and $${\rm{\Delta }}{l}_{2}$$ are the corresponding correction lengths expressed as

5 $${\rm{\Delta }}{l}_{1}={\rm{0.82}}[{\rm{1}}-{\rm{1.35}}\frac{{r}_{{\rm{n}}}}{{r}_{{\rm{c}}}}+{\rm{0.31}}{(\frac{{r}_{{\rm{n}}}}{{r}_{{\rm{c}}}})}^{3}]{r}_{{\rm{n}}},$$

6 $${\rm{\Delta }}{l}_{2}={\rm{0.82}}\,[{\rm{1}}-{\rm{0.235}}\frac{{r}_{{\rm{n}}}}{{r}_{{\rm{t}}}}-{\rm{1.32}}{(\frac{{r}_{{\rm{n}}}}{{r}_{{\rm{t}}}})}^{2}+{\rm{1.54}}{(\frac{{r}_{{\rm{n}}}}{{r}_{{\rm{t}}}})}^{3}-{\rm{0.86}}{(\frac{{r}_{{\rm{n}}}}{{r}_{{\rm{t}}}})}^{4}]{r}_{{\rm{n}}},$$

Considering the sound velocity at the end of MHR is ${v}_{(x={\rm{end}})}={\rm{0}}$, the impedance at the neck of the MHR can be derived as

7 $${Z}_{{\rm{MHR}}}={M}_{{\rm{total}}}(1,1)/{M}_{{\rm{total}}}(2,1){\rm{.}}$$

Therefore, we characterize the MHR with effective impedance surface to conduct theoretical analysis in this work.

### Experimental measurements

The experimental MHR samples are fabricated with epoxy resin (mass density ${\rho }_{e}={\rm{1050}}\,{\rm{kg}}/{m}^{3}$ and longitudinal sound velocity ${c}_{e}={\rm{2200}}\,{\rm{m}}/{\rm{s}}$) by 3D printing technology, with a wall thicknesses of 3 mm. The straight waveguide with side thickness 8 mm is made by PMMA. Four 1/4 inch condensed microphones (Brüel & Kjær type-4939) have been used to record the sound pressure information. The experimental measurements are conducted by the two-load method^19^ in the standard test method ASTM E2611-09, where hard wall and acoustic sponge (as anechoic boundary) are separately used at the terminal.

## Data Availability

The datasets generated during and/or analyzed during the current study are available from the corresponding author on reasonable request.

## References

[1] Cheng Y, Xu JY, Liu XJ (2008). One-dimensional structured ultrasonic metamaterials with simultaneously negative dynamic density and modulus. Phy. Rev. B.

[2] Fang N (2006). Ultrasonic metamaterials with negative modulus. Nat. Mater..

[3] Li Y (2017). Tunable Asymmetric Transmission via Lossy Acoustic Metasurfaces. Phys. Rev. Lett..

[4] Jiménez N, Cox TJ, Romero-García V, Groby J-P (2017). Metadiffusers: Deep-subwavelength sound diffusers. Sci. Rep..

[5] Yang X, Yin J, Yu G, Peng L, Wang N (2015). Acoustic superlens using Helmholtz-resonator-based metamaterials. Appl. Phys. Lett..

[6] Xia J-P (2018). Broadband Tunable Acoustic Asymmetric Focusing Lens from Dual-Layer Metasurfaces. Phys. Rev. Appl..

[7] Groby J-P (2015). Enhancing the absorption properties of acoustic porous plates by periodically embedding Helmholtz resonators. J. Acoust. Soc. Am..

[8] Romero-García V (2016). Perfect and broadband acoustic absorption by critically coupled sub-wavelength resonators. Sci. Rep..

[9] Romero-García V, Theocharis G, Richoux O, Pagneux V (2016). Use of complex frequency plane to design broadband and sub-wavelength absorbers. J. Acoust. Soc. Am..

[10] Merkel A, Theocharis G, Richoux O, Romero-García V, Pagneux V (2015). Control of acoustic absorption in one-dimensional scattering by resonant scatterers. Appl. Phys. Lett..

[11] Wei P, Croënne C, Chu ST, Li J (2014). Symmetrical and anti-symmetrical coherent perfect absorption for acoustic waves. Appl. Phys. Lett..

[12] Li J, Wang W, Xie Y, Popa B-I, Cummer SA (2016). A sound absorbing metasurface with coupled resonators. Appl. Phys. Lett..

[13] Jiménez N, Huang W, Romero-García V, Pagneux V, Groby J-P (2016). Ultra-thin metamaterial for perfect and quasi-omnidirectional sound absorption. Appl. Phys. Lett..

[14] Cummer SA, Christensen J, Alù A (2016). Controlling sound with acoustic metamaterials. Nat. Rev. Mater..

[15] Ryoo H, Jeon W (2018). Dual-frequency sound-absorbing metasurface based on visco-thermal effects with frequency dependence. J. Appl. Phys..

[16] Baz A (2009). The structure of an active acoustic metamaterial with tunable effective density. New J. Phys..

[17] Ma G, Yang M, Xiao S, Yang Z, Sheng P (2014). Acoustic metasurface with hybrid resonances. Nat Mater.

[18] Fu C, Zhang X, Yang M, Xiao S, Yang Z (2017). Hybrid membrane resonators for multiple frequency asymmetric absorption and reflection in large waveguide. Appl. Phys. Lett..

[19] Long H, Cheng Y, Liu X (2017). Asymmetric absorber with multiband and broadband for low-frequency sound. Appl. Phys. Lett..

[20] Jiménez N, Romero-García V, Pagneux V, Groby J-P (2017). Rainbow-trapping absorbers: Broadband, perfect and asymmetric sound absorption by subwavelength panels for transmission problems. Sci. Rep..

[21] Stinson MR (1991). The propagation of plane sound waves in narrow and wide circular tubes, and generalization to uniform tubes of arbitrary cross‐sectional shape. J. Acoust. Soc. Am..

[22] Richoux O, Pagneux V (2002). Acoustic characterization of the Hofstadter butterfly with resonant scatterers. EPL (Europhysics Letters).

[23] Liu F (2010). Acoustic analog of electromagnetically induced transparency in periodic arrays of square rods. Phys. Rev. E.

[24] Yang M, Sheng P (2017). Sound Absorption Structures: From Porous Media to Acoustic Metamaterials. Annu. Rev. Mater. Res..

[25] Mei J (2012). Dark acoustic metamaterials as super absorbers for low-frequency sound. Nat. Commun..

[26] Yang M (2015). Subwavelength total acoustic absorption with degenerate resonators. Appl. Phys. Lett..

[27] Li Y, Assouar BM (2016). Acoustic metasurface-based perfect absorber with deep subwavelength thickness. Appl. Phys. Lett..

[28] Cai X, Guo Q, Hu G, Yang J (2014). Ultrathin low-frequency sound absorbing panels based on coplanar spiral tubes or coplanar Helmholtz resonators. Appl. Phys. Lett..

[29] Wu X (2016). Low-frequency tunable acoustic absorber based on split tube resonators. Appl. Phys. Lett..

[30] Long H, Cheng Y, Tao J, Liu X (2017). Perfect absorption of low-frequency sound waves by critically coupled subwavelength resonant system. Appl. Phys. Lett..

[31] Long H, Gao S, Cheng Y, Liu X (2018). Multiband quasi-perfect low-frequency sound absorber based on double-channel Mie resonator. Appl. Phys. Lett..

[32] Zhang C, Hu X (2016). Three-Dimensional Single-Port Labyrinthine Acoustic Metamaterial: Perfect Absorption with Large Bandwidth and Tunability. Phys. Rev. Appl..

[33] Yang M, Chen S, Fu C, Sheng P (2017). Optimal sound-absorbing structures. Mater. Horiz..

[34] Meng C, Zhang X, Tang ST, Yang M, Yang Z (2017). Acoustic Coherent Perfect Absorbers as Sensitive Null Detectors. Sci. Rep..

[35] Zhang X, Meng C, Yang Z (2017). Wave Manipulations by Coherent Perfect Channeling. Sci. Rep..

[36] Huo S, Chen J, Huang H, Huang G (2017). Simultaneous multi-band valley-protected topological edge states of shear vertical wave in two-dimensional phononic crystals with veins. Sci. Rep..

[37] Feng L, Huang H, Zhang J, Xie X, Chen J (2018). Reconfigurable topological phononic crystal slabs. Phys. Lett. A.

